# Characterization and Performance Evaluation of Cellulose Acetate–Polyurethane Film for Lead II Ion Removal

**DOI:** 10.3390/polym12061317

**Published:** 2020-06-09

**Authors:** M. Iqhrammullah, Marlina Marlina, H. P. S. Abdul Khalil, K. H. Kurniawan, H. Suyanto, R. Hedwig, I. Karnadi, N. G. Olaiya, C. K. Abdullah, S. N. Abdulmadjid

**Affiliations:** 1 Graduate School of Mathematics and Applied Sciences, Universitas Syiah Kuala, Banda Aceh 23111, Indonesia; m.iqhram@oia.unsyiah.ac.id; 2 Department of Chemistry, Faculty of Mathematics and Natural Sciences, Universitas Syiah Kuala, Banda Aceh 23111, Indonesia; marlina@unsyiah.ac.id; 3 School of Industrial Technology, Universiti Sains Malaysia, Penang 11800, Malaysia; ck_abdullah@usm.my; 4 Research Center of Maju Makmur Mandiri Foundation, 40/80 Srengseng Raya, Jakarta 11630, Indonesia; kurnia18@cbn.net.id; 5 Faculty of Mathematics and Natural Sciences, Udayana University, Kampus Bukit Jimbaran, Denpasar 80361, Bali, Indonesia; hery6@yahoo.com; 6 Department of Computer Engineering, Faculty of Engineering, Bina Nusantara University, Jakarta 11480, Indonesia; rinda@binus.edu; 7 Department of Electrical Engineering, Krida Wacana Christian University, Jakarta 11470, Indonesia; indra.karnadi@ukrida.ac.id; 8 Department of Industrial and Production Engineering, Federal University of Technology, PMB 704, Akure 340252, Ondo State, Nigeria; ngolaiya@futa.edu.ng; 9 Department of Physics, Faculty of Mathematics and Natural Sciences Universitas Syiah Kuala, Banda Aceh 23111, Indonesia

**Keywords:** cellulose acetate, polyurethane, film, adsorbent, environmental

## Abstract

Global pollution from toxic metal waste has resulted in increased research on toxic metal adsorption. A cellulose acetate–polyurethane (CA–PU) film adsorbent was successfully prepared in this research. The cellulose acetate–polyurethane film adsorbent was prepared with a polycondensation reaction between cellulose acetate and methylene diphenyl diisocyanate. The CA–PU bond formation was confirmed by functional group analysis obtained from Fourier transform infrared (FTIR) spectroscopy. The obtained film was characterized for improved tensile and thermal properties with the addition of methylene diphenyl diisocyanate (MDI). The adsorption ability of the obtained film was evaluated with laser-induced breakdown spectroscopy (LIBS). The best film adsorbent from the LIBS was selected and studied for adsorption isotherm. The FTIR analysis confirmed the formation of the CA–PU bond from the polycondensation between cellulose acetate and the methylene diphenyl diisocyanate. The result showed that the addition of methylene diphenyl diisocyanate (MDI) resulted in the urethane network’s growth. The characterization result showed an improvement in the morphology, thermal stability, and tensile strength of the film. The LIBS studies showed improvement in the adsorption of Pb^2+^ with CA–PU compared with the neat CA. The isotherm studies revealed that Pb^2+^ adsorption by cellulose acetate–polyurethane film adsorbent was heterogeneously dependent on the Freundlich isotherm model (R^2^ = 0.97044). Overall, the polycondensation method proposed by this study enhanced the Pb^2+^ removal, and was comparable to those reported in previous studies.

## 1. Introduction

The adsorption of heavy toxic metals to prevent environmental pollution has become a global issue. The global pollution from toxic metals released from industrial wastewater and mining sites has been on the increase. The increase in the pollution caused by toxic metals released has resulted in the development of more efficient and effective materials as adsorbents. Biopolymers have been the focus of intensive study on its application in packaging and biomedical materials. More recently, polymeric films from biopolymers have been proposed as a good adsorbent for toxic heavy metals [1,2].

Cellulose has been reported as the most abundant polysaccharide polymer on earth and has been used for several industrial applications. Many studies have attempted to improve on the multifunctionality of cellulose through a reaction with various reagents. In order to achieve this, the three (3) hydroxyl (OH) groups in the cellulose anhydroglucose unit, in its dissolved state, reacted with the acetic acid and acetic anhydrate to produce cellulose acetate. This cellulosic derivative was found to have better durability than native cellulose. Previous research has established the solubility of cellulose acetate (with substitution degree >2) in common solvents such as tetrahydrofuran, methyl acetate, acetone, and dioxane [3]. The solubility of cellulose acetate in these solvents enhances its ability to be formed into different shapes through the phase inversion technique. As a result of these properties, cellulose acetate has been used as a film-shaped adsorbent for heavy metal pollution such as lead (Pb) [4].

Lead (Pb) has been described as a toxic heavy metal from industrial waste and mining sites. Lead (Pb) has aroused global concern because of its environmental pollution. El Azhari et al. [5] reported the Pb pollution in a former mining area in Morocco. The worse condition was reported by Islam et al. [6] on Pb pollution in Bangladesh’s urban river area. Moderate to high Pb contamination was found in food resources (fish and oyster), collected from Meiliang Bay of Taihu Lake in China [7]. Pollution and contamination caused by Pb deposits have also been reported in Indonesia [8] and India [9]. Pb exposure, either through ingestion from contaminated foods or direct contact, can devastate human health [10]. It has been predicted that Pb pollution will increase with urbanization and industrialization [11]. The sporadic increase in the reported cases has been of major concern to researchers and is a global threat to human health.

Adsorption has been proposed as a possible solution for the pollution problem caused by toxic heavy metals. It has also been suggested as a way out for the industrial wastewater management of heavy metal release to the environment. Adsorbents have been produced from a wide variety of materials such as biochar [12], Mxenes [13], graphene oxide (GO), and metal–organic framework (MOF) [14]. Compared to other methods such as electrocoagulation, membrane filtration, reverse osmosis, etc., adsorption has been reported as being more economical and efficient. In particular, biomass-based adsorbents have gained research interest due to its sustainability [15,16]. Cellulose (and its derivatives) from biomass has been proposed as a material to produce a toxic heavy metal adsorbent [4,16,17,18]. Previous studies had been conducted to improve the sturdiness of cellulose-based material. Salama et al. [16] produced a cellulose acetate/carbon nanotube composite and reported an improvement in tensile strength. Similar improvements in tensile strength and thermal stability were reported in previous studies with the blending method [19,20,21].

The report of Kumari et al. [22] on the synthesis of a cellulose nanowhisker-based polyurethane foam suggests that the NH and C=O functional groups obtained from the polycondensation contributed to the cationic organic pollutant adsorbent. Their work proposed the use of CA–PU for the removal of Pb^2+^ ions. Based on the explanation above, this study produced a cellulose acetate–polyurethane film with a polycondensation method and explored its application for Pb^2+^ ion removal from an aqueous solution. In this research, the polycondensation reaction produced a polyurethane linkage, which enhanced the adsorption properties of the CA–PU film. However, the use of cellulosic materials in polyurethane preparation has not been fully explored. Furthermore, previous studies on the use of cellulose derivatives used the blending method as opposed to the polymer condensation method used in this study [19,22,23].

## 2. Materials and Methods

### 2.1. Materials

The materials used in this research included cellulose acetate (CA) (39.3 wt% polymer acetyl), methylene diphenyl diisocyanate (MDI), 1,4-dioxane, lead (II), NaOH, KOH, HNO_3_, and lead (II) acetate (Pb(CH_3_COO)_2_). The analytical grades of the materials were purchased from Sigma-Aldrich (Selangor, Malaysia).

### 2.2. Preparation of Cellulose Acetate–Polyurethane (CA–PU) Film

Methylene diphenyl diisocyanate (MDI) were used for the preparation of polyurethane without the addition of a catalyst. The preparation of the CA–PU film was conducted with a modified procedure adapted from the reports of Riaz et al. [19] and Li et al. [23]. A combination of polycondensation and phase inversion technique was used in the preparation. The polycondensation reaction was used to prepare the film, while the phase inversion technique was used to control the transformation of the solution to a solid-state. CA powder was weighed as much as 1 g and dissolved into 10 mL 1,4-dioxane. The mixture was heated to 100 °C and stirred at 1000 rpm. After 15 min, the homogenous mixed solution stirring speed was reduced to 250 rpm, followed by the dropwise addition of MDI with the composition presented in Table 1. The polymer mixture was stirred for another 60 s after the addition of MDI and poured into a silicon oil-coated mold. The polymer mix was left in the silicon oil-coated mold and oven-dried at room temperature for 24 h to form a film. Cellulose acetate (CA) was dissolved in 1,4-dioxane to form a CA film and used as the control film. However, for the neat CA film, the cast solution was left for 72 h to obtain the film.

**Figure 1:**
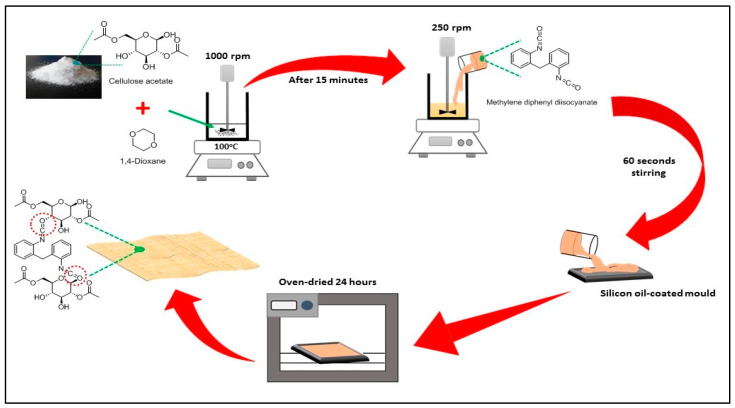
The preparation process of neat cellulose acetate (CA) and cellulose acetate–polyurethane (CA–PU) films.

The film was removed from the mold and dipped in 50 mL acetone for 15 min to remove the excess MDI. The film was then washed with 2% NaOH for 60 min, and with distilled water several times until a neutral pH was obtained. The drying process was carried out in an oven vacuum at 70 °C for 12 h. The obtained CA–PU film was cut to 1 × 1 cm^2^ and stored in a zip-lock bag for characterization. Figure 1 shows the step-by-step preparation process of the neat CA and CA–PU films.

### 2.3. Characterization of the Film

FTIR analysis was conducted on the obtained film to establish the formation of CA–PU bonding. Functional group analysis of CA–PU was carried out with Fourier transform infrared (FTIR) (Shimadzu FT-IR—Prestige 21 Series, Shimadzu, Kyoto, Japan). The samples were cut into standard sizes and oven-dried at 60 °C overnight before the test. The test was conducted in transmittance mode. The wettability of the film was studied with the contact angle measurement. The contact angle was measured with a KSV CAM 10 (KSV Instruments Ltd., Espoo, Finland) at a test liquid speed of 5 fps, and the sample snapped angle were recorded. The average of the angle was calculated, and the standard error reported

The mechanical strength was characterized by a Universal Testing Machine HT8503 at ASTM D638 to observe the tensile strength of the obtained film. The morphology of the obtained film was studied to observe possible agglomeration from unreacted CA or the methylene diphenyl diisocyanate (MDI). The samples were coated to improve its conduction and observed under scanning electron microscopy (SEM) (Jeol. Jsm-6510 LA, Joel, Tokyo, Japan) with 4000× magnification at 20 kV. The three-dimensional view of the film topography was observed with atomic force microscopy (AFM). The film surface AFM images were captured with an AFM XE-70 park system. The samples were cut into 2 cm × 2 cm dimensions, and the image captured at Z servo gain of 2.5, scan area 30 µm, scan rate 0.3 Hz, and setpoint 14 nm.

The thermal properties of the control and CA–PU films were studied with thermogravimetry analysis (TGA) and differential scanning calorimetry (DSC). The thermogravimetry analysis and derivative thermogravimetry analysis values (TGA-DTA) of the CA–PU films were analyzed with a Shimadzu DTG-60 thermal gravimetric analyzer (Shimadzu, Kyoto, Japan) with ambient nitrogen (flow rate 20 mL/min.) and a temperature rate of 10 °C/min. Furthermore, the differential scanning calorimetry (DSC) was studied to observe the melting and crystallization curve of the obtained film. The DSC was conducted with a Shimadzu DSC-60 differential scanning calorimeter under ambient nitrogen (flow rate 30 mL/min.) run at 50–600 °C.

### 2.4. Batch Adsorption Performance Evaluation of the Film

The Pb^2+^ adsorption on CA and CA–PUs was qualitatively analyzed with laser-induced breakdown spectroscopy (LIBS). The adsorption was studied with a batch method. First, 1.57 g of Pb (CH_3_COO)_2_ was dissolved in 1000 mL of distilled water to obtain a 1000 mg/L Pb^2+^ stock solution. It was further diluted into 200 mg/L with distilled water. The film adsorbents were inserted into a 100 mL Erlenmeyer flask filled with 25 mL Pb^2+^ 200 mg/L solution for 12 h at pH 5.5. The Erlenmeyer was placed on the shaker at 450 rpm to ensure the solution’s homogenous distribution of the Pb ion. Afterward, the adsorbent was removed from the solution and left to dry. The sample was then shot with a Nd-YAG Laser (Quanta Ray, LAB SERIES, Spectra - Physics, Santa Clara, USA, 1064 nm, 450 mJ, 8 ns). LIBS analysis was carried out under an air atmosphere with a 1000 ns delay time and energy of 54 mJ. Further studies were conducted on the effect of the solution’s contact time with the adsorbent, pH of the solution, and isotherm studies. Additionally, for pH adjustment, KOH was used to increase the pH, and HNO_3_ was used to decrease the pH. The pH of the Pb^2+^ solution was measured with an EC500 pH-meter. The batch adsorption was carried out with a variation in the initial concentration between 10 and 200 mg/L. Pb^2+^ concentration after the adsorption was quantified with atomic absorbance spectroscopy (AAS) (Shandon Southern A3400 Runcorn, Cheshire, UK) [24]. The adsorption capacity at *t* time (*q_t_*) (mg/g) and removal percentage were calculated with Equations (1) and (2), respectively.

(1) $$q_{t}=\frac{C_{0}-C_{t}}{W}\times V$$

(2) $$\;\%\mathrm{Removal}=\frac{C_{0}-C_{t}}{C_{0}}\times 100\%\;$$

where *C*_0_ and *C_t_* are concentrations at 0 and *t* time (ml/L), respectively; *W* is the adsorbent weight (g); and *V,* the Pb^2+^ volume (L).

## 3. Results and Discussion

### 3.1. Reaction and Functional Group Analysis

**Figure 2:**
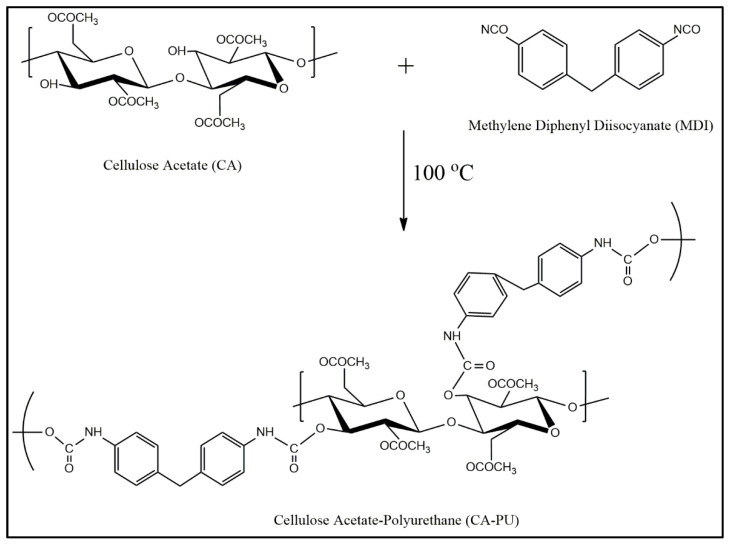
Cellulose acetate and methylene diphenyl diisocyanate (MDI) was reacted to produce the cellulose acetate–polyurethane (CA–PU) film.

The schematic diagram of the chemical reaction of cellulose acetate (CA) and isocyanate is presented in Figure 2. Cellulose acetate–polyurethane (CA–PU) film was obtained through a condensation reaction between the hydroxyl group from cellulose anhydroglucose units and the isocyanate group from methylene diphenyl diisocyanate (MDI). This reaction formed a urethane linkage. This reaction was adopted from the work of Góes et al. [25] and Ikhwan et al. [26], who synthesized cellulose-based hybrid polyurethane products. Rivera-Armenta et al. [27] also produced a polyurethane foam that was modified with various types of cellulose derivates with the condensation reaction. Cellulose acetate was dissolved in 1,4-dioxane at 100 °C, and the solution was used in the polycondensation reaction with MDI, as proposed by Fischer et al. [21]. The dissolution procedure resulted in the reaction of the hydroxyl group from cellulose anhydroglucose units with the isocyanate to form the CA–PU film.

**Figure 3:**
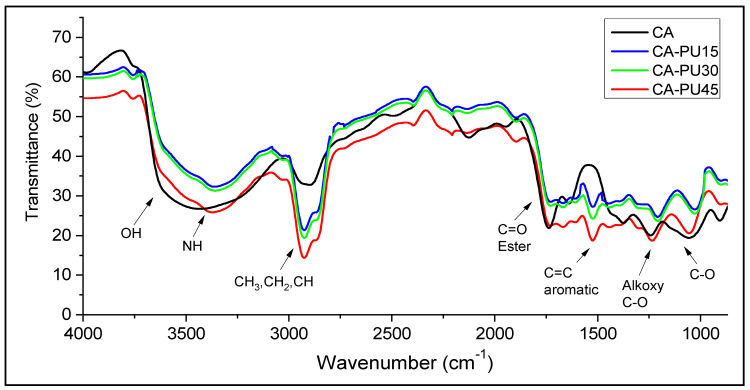
Fourier transform infrared (FTIR) spectra of CA, CA–PU15, CA–PU30, and CA–PU.

The formation of a urethane linkage through OH and isocyanate condensation was confirmed by the FTIR analysis, as presented in Figure 3. The formation of the urethane linkage was observed in the OH stretched band of the FT-IR spectra at 3633–3532 cm^−1^. The hydrogen bond (OH) was observed to increase with the percentage of CA content and vice versa with the addition of MDI. The formation of NH was observed at 3379 cm^−1,^ and this was attributed to the condensation between the OH and CNO groups [28]. A similar bond was observed in the FTIR report of CA–PU by Rivera-Armenta et al. [27] and Ikhwan et al. [26]. The C=C aromatic rings observed at 1519 cm^−1^ was formed from the introduction of MDI in the reaction. The MDI also introduced a high absorbance of CH stretched vibration at 2927 cm^−1^. The number of introduced aromatic rings was affected by the percentage of the added MDI, as indicated by the lower absorbance intensities in CA–PU15 and CA–PU30 compared to CA–PU45.

Wavenumber shifts were observed at 2927 to 2900 cm^−1^ due to the disturbance from the introduction of new functional groups. The wavenumber shift in the new materials was also observed at 1261 to 1266 cm^−1^, which were assigned to the bending vibrations of the C–O alkoxy. Other typical functional groups were observed in the stretched vibration absorbances at 1735 cm^−1^ for C=O and 1053 cm^−1^ for C–O. These functional groups, formed by a carbon bond with oxygen (C=O and C–O), have been reported to be responsible for the pollutant uptake by cellulose-based adsorbents [29,30,31,32]. A similar result was reported in previous studies that confirmed the formation of a urethane linkage with a band stretch at 3330 cm^−1^ overlapped with OH stretching [31,33]. The extensive formation of urethane linkages was confirmed by their characteristic bands that were apparent at 3330 cm^−1^, which appears to overlap the O–H stretching vibration. Similar FTIR results were obtained by Tenorio-Alfonso [33].

The wettability analysis further confirmed the reaction between the OH from the cellulose acetate and the isocyanate. Table 2 shows the results of the wettability test of the control and CA–PU film with contact value. The values of the contact angle ranged from 38.2° to 62.5°. The contact value of the CA was observed to be lower when compared to the CA–PU films. Based on these values, the films’ wettability properties tended toward hydrophobia with the addition of PU. The observed increase in the contact angle can be explained by the nature of the two materials (i.e., cellulose acetate with hydrophilic nature and polyurethane with hydrophobic nature) [34]. The average value of the contact angle measured showed that the addition of polyurethane to cellulose acetate to form a cellulose acetate–polyurethane film improved the hydrophobic properties of cellulose acetate. The increase in the hydrophobic properties of the film formed resulted in better water-resistant properties [35]. The hydrophobic properties increased with the addition of a higher percentage of polyurethane. Based on the observations in the wettability analysis, the adsorption of water by the CA changed it to a gel-like material once immersed in water, while CA–PU maintained its rigidity when in the water. This observation confirmed the result of the contact angle that CA–PU was more hydrophobic than hydrophilic. 

The behavior of the films with water was the reaction of the isocyanate with OH groups in the CA, which resulted in the loss of its OH groups (confirmed by FTIR spectra). The OH group was responsible for the hydrophilicity since it formed bonds with water molecules. Therefore, the loss of OH groups in CA molecules due to the reaction with isocyanate caused the CA–PU film to be more hydrophobic

### 3.2. Mechanical Properties

Table 3 reports the results of the mechanical properties of the neat CA and CA–PU films. As seen in the table, the tensile strength increased with the addition of MDI. This suggests a direct correlation between MDI addition and the mechanical properties of the CA–PU film. The initial addition of 15% MDI only increased the tensile strength by 0.2 MPa. Thus, the films’ mechanical properties were affected by the adhesion and cohesion of the polymer constituents [36]. 

This insignificant increase in the tensile strength was possibly due to the inhomogeneous mix of CA–PU15. CA–PU15 had an overwhelming percentage of cellulose acetate, and this prevented the even distribution of the urethane linkage network, hence, a reduction in the homogeneity of the film. Consequently, the cohesive force between the CA molecules was observed to be prevalent and resulted in a weak tensile strength. The increase in the MDI percentage from 15 to 30% significantly increased the tensile strength by 2.9 MPa. This trend was observed with an increase in the percentage of MDI up to 45%. At the addition of 45% MDI, the number of urethane linkage networks formed increased and were evenly distributed in the material. The CA–PU films’ increased mechanical properties confirmed the bond formed between the –OH and isocyanate, as observed in the FTIR results.

**Figure 4:**
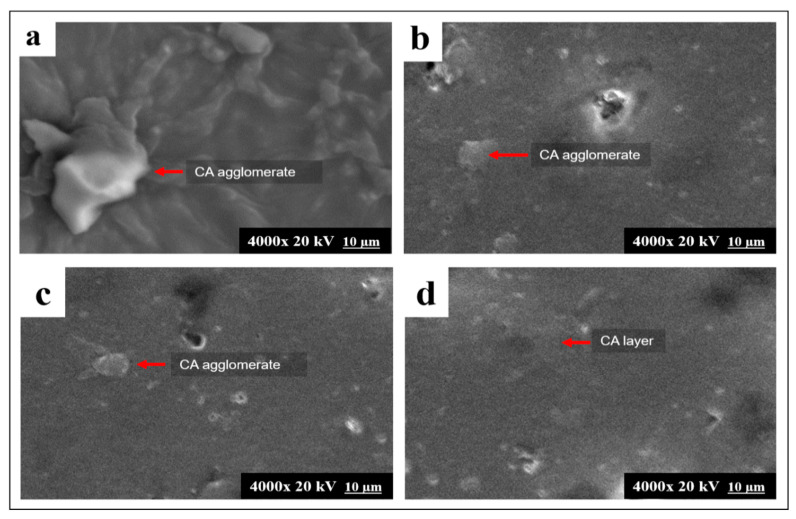
Scanning electron microscope (SEM) images of (**a**) neat CA; (**b**) CA–PU15; (**c**) CA–PU30 and (**d**) CA–PU45.

The result of the morphological analysis corroborated the tensile properties of the CA and CA–PU films. Figure 4 shows the scanning electron microscopy (SEM) images of the surface morphology of the CA and CA–PU samples. The cellulose acetate film was observed to form agglomerates on the surface, and this was probably due to the intermolecular interaction of hydrogen bonding present in the cellulose acetate molecules. However, no agglomeration was observed with 45% MDI. This showed that the agglomerates observed with 15 to 30% of MDI were due to an insufficient MDI percentage. The observation from the morphology images from SEM can be explained with the result of the FTIR analysis. The FTIR analysis showed the formation of the urethane linkage, which reduced the availability of the hydroxyl groups for hydrogen bond interaction [37]. The reduction in the hydrogen bonds freed the molecules for intermolecular interaction. The intermolecular attraction induced between similar molecules resulted in the formation of the agglomerate [38].

Furthermore, the significant difference between the time required by the solution cast of CA–PUs and the neat cellulose acetate was due to the intermolecular bond. The CA–PU cast dried formed a film within 24 h, while the neat cellulose acetate took at least 72 h to dry. The time-rate difference showed that the CA–PU molecules were in a locked position in the mixture due to the combined effect of the hydrogen bond and other chemical urethane linkages between CA and PU.

The formation of agglomerates reduced the binding sites of the adsorbent film and practically increased the pollutant removal. Nevertheless, in CA–PU, heterogeneous layers of CA were observed, which indicated the presence of bulk CA molecules. Furthermore, the CA–PU film surface appeared to have porous-like holes, which indicated a higher contact surface of the adsorbent. Hence, CA–PU had greater adsorption enhanced morphology compared to the cellulose acetate film. 

**Figure 5:**
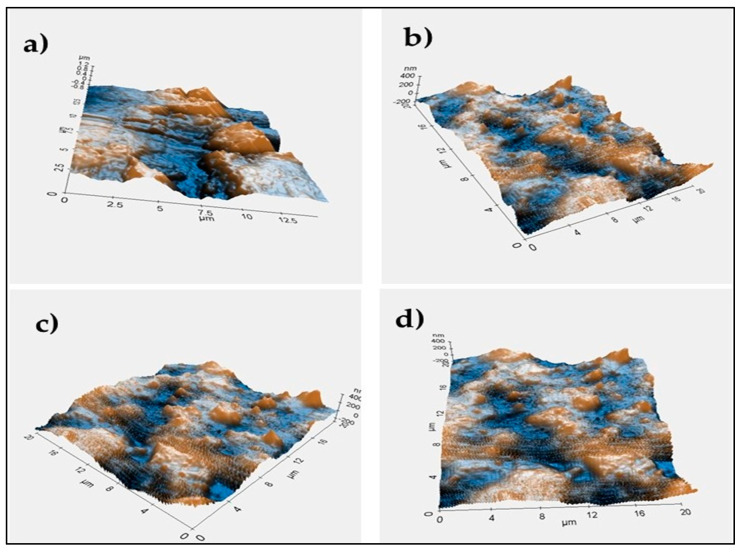
Atomic force microscopy of the (**a**) neat CA; (**b**) CA–PU15; (**c**) CA–PU30, and (**d**) CA–PU45.

The result of the AFM images of the neat CA and CA–PU films are presented in Figure 5. The neat CA was observed to be a less solid form with undeveloped undulated patterns when compared to the CA–PU samples. The topography height of the AFM images was observed to increase with the addition of MDI. The result of the AFM further corroborated the agglomeration observed at 15%, CA–PU, which further reduced with an increase in the quantity of MDI to 45%. The AFM images in Figure 5 show a rough surface topography with a lower percentage of MDI and a smoother surface with a higher percentage of MDI. The AFM images showed that an even distribution of MDI increased up to 45% [39]. The even distribution of the MDI in CA increased the topography height, and the increased thickness of the AFM images with the addition of MDI further justified the increased tensile strength observed in the results presented in Table 3 [40]. The change in the roughness of the AFM 3D topographical images confirmed the interaction between CA and MDI. The interaction was confirmed from the FTIR results as a chemical bond between CA and MDI. 

### 3.3. Thermal Properties

**Figure 6:**
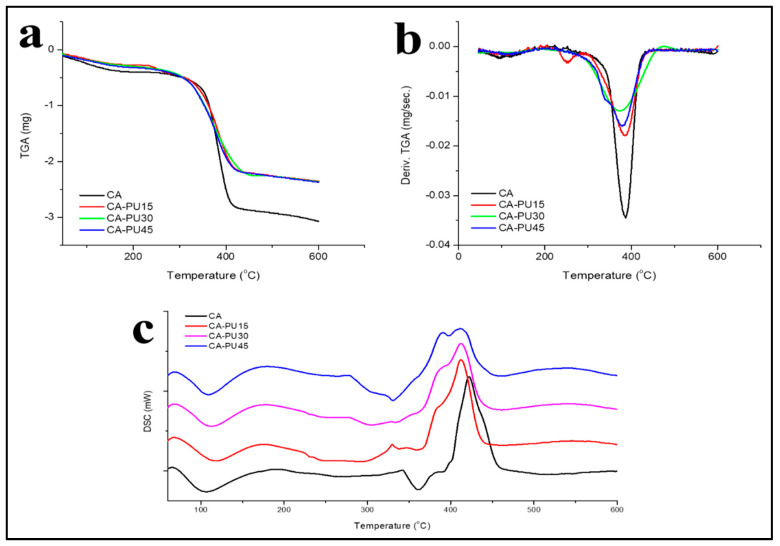
(**a**) TGA; (**b**) DTGA; and (**c**) DSC thermogram of CA–PU15, CA–PU30, CA–PU45, and CA.

The thermal gravimetry analysis (TGA) degradation profile of CA and CA–PUs is presented in Figure 6a and its derivative (DTGA) thermogravimetric in Figure 6b. Initial mass degradation at around 60–165 °C was observed, which was due to the removal of volatile contents such as the solvent used or water. The slight degradation was confirmed by the endothermic change in the DSC graph (Figure 6c). However, the glass transition temperature (Tg) of the film’s initial volatile content could not be observed.

It was observed in Figure 6b that the neat CA film had a single degradation peak (T_max_ = 386 °C), while for the CA–PUs, degradation appeared to have multiple peaks. A distinct peak at 250 °C in CA–PU15 can be attributed to the degradation of the urethane linkage [41]. In CA–PU45, this peak was joined with the cellulose acetate degradation due to the expansion of the urethane network that became more homogenous. The report by Riaz et al. [19], stated that the degradation of urethane linkages contributed more to the thermal stability properties as the heat may not directly affect the cellulose acetate. The improvement in thermal stability was observed in the positive shift of the DTGA peaks and the increased residue at 450 °C, where cellulose acetate had a 0.21 mg residue, and all CA–PUs had around 0.78 mg.

The thermal properties of the cellulose acetate and CA–PUs were further analyzed with DSC (Figure 6c). The crystallization temperatures (*T_c_*) of the films ranged from 170 to 185 °C and melting temperatures (*T_m_*) between 330 and 350 °C. These values were similar to those reported in previous research [33]. The degradation temperature (*T_d_*) of urethane linkage shifted and overlapped that of the *T_d_* of cellulose acetate. Greater formation of urethane linkages (due to the increase in MDI addition) contributed to the resemblance of the decomposition behaviors between the urethane and cellulose acetate. Thus, it was inferred that the increase in the homogenous distribution of the urethane linkage network occurred within the CA–PU film. The sequential increased shift in the degradation temperature (*T_d_*) peak of CA–PU15, CA–PU30, and CA–PU45 showed increased thermal stability of the urethane network.

### 3.4. Pb^2+^ Ions Adsorption Detected with Laser-Induced Breakdown Spectroscopy (LIBS)

**Figure 7:**
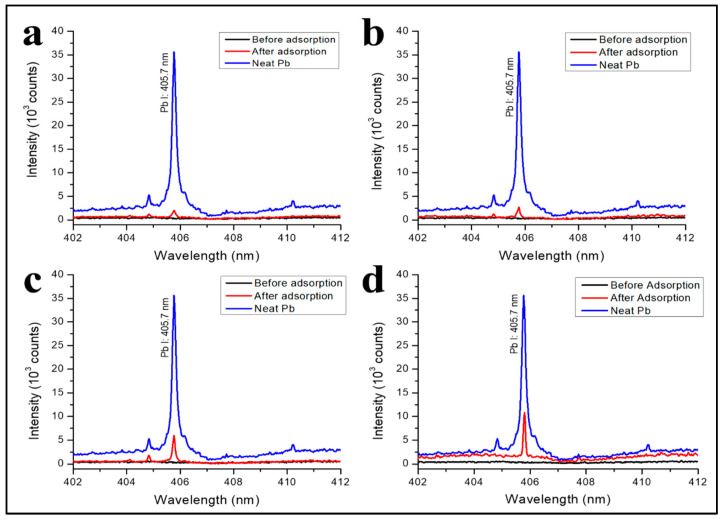
LIBS spectra of (**a**) CA; (**b**) CA–PU15; (**c**) CA–PU30; and (**d**) CA–PU45 before and after the adsorption, in comparison with the neat Pb spectra.

Pb^2+^ adsorption on the CA and CA–PU surface were qualitatively analysed with LIBS in an air atmosphere (1 atm) with the delay time of 1 µs. The spectra obtained from the LIBS analysis are presented in Figure 7. The emission intensity of Pb I 405.7 nm confirmed the Pb^2+^ enrichment on the CA–PU surface. As shown in Figure 7, all CA–PUs before the adsorption produced flat emission spectra at 405.7 nm and indicated the absence of Pb. After the adsorption, the Pb line was observed and compared to the spectral line given by the neat Pb. The adsorption of Pb^2+^ by CA–PU was detected in the LIBS analysis and further confirmed its application in the heavy metal qualitative analysis [42]. The enhancement of Pb’s adsorption by the CA–PU compared with the neat CA was probably due to the introduction of N- and O-containing functional groups from the polymer condensation reaction. 

**Figure 8:**
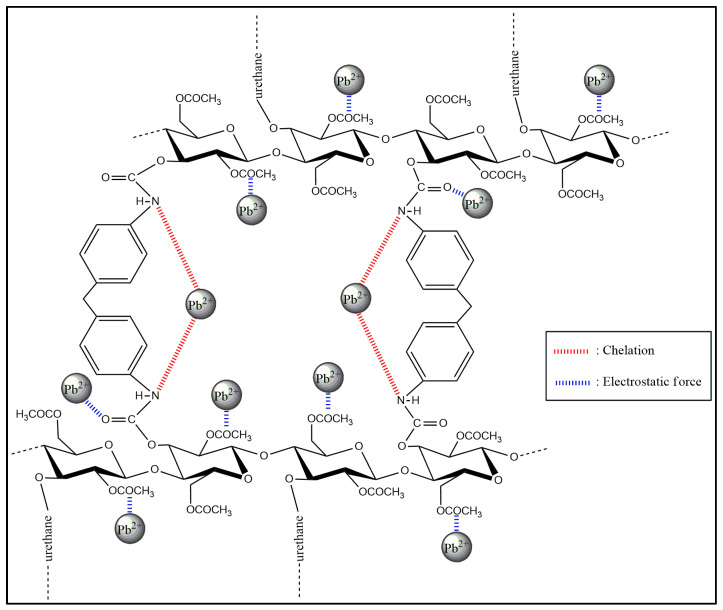
Proposed Pb^2+^ adsorption mechanism on the CA–PU film.

The spectral intensity was used to qualitatively estimate the number of adsorbed Pb^2+^ ions on the surface of the CA–PU. Compared with other methods, this method took less time and required no pre-treatment. In addition, LIBS exclusively detected the adsorbed Pb^2+^ enriched on the surface of the adsorbent. The spectra showed that the emission intensity of Pb I 405.7 nm was produced from CA and CA–PU15 even though that of the latter adsorbent was a little bit higher. The results of the LIBS analysis proved the enhancement of binding sites after the polycondensation, which was also reported by Kumari et al. [22]. Pb^2+^ adsorption of CA–PU45 was due to OH, CO, and NH functional groups, which acted as the binding sites [43]. Previous studies reported the adsorbate–adsorbent interaction through the electrostatic force [44,45]. Some others have reported that the chelation is responsible for the mechanism of Pb uptake, and the N-containing functional groups (such as NH and N-C=O) serve as the ligand [12,24]. The proposed mechanism for Pb^2+^ adsorption on the prepared CA–PU in this research is presented in Figure 8.

The intensity further increased sequentially in CA–PU30 and CA–PU45. Aside from the functional groups, the morphology of CA–PU also contributed to the Pb^2+^ adsorption, as confirmed by the SEM analysis. From the analysis, it can be seen that CA–PU45 had more contact surface because the agglomerate formation was inhibited. As a result, more binding sites were available for the adsorbent to interact with. This qualitative investigation with LIBS suggests that CA–PU45 was the best film adsorbent. Thus, CA–PU45 was used for further studies.

### 3.5. Effect of Contact Time, pH, and Initial Concentration

**Figure 9:**
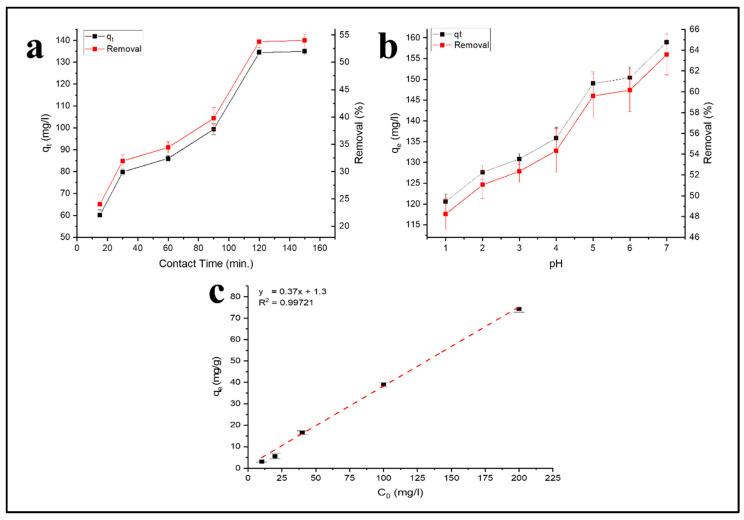
Effect of (**a**) contact time; (**b**) pH; and (**c**) initial concentration on Pb^2+^ removal by the CA–PU film adsorbent.

Figure 9 shows the effect of contact time and solution acidity (pH) on Pb^2+^ ion uptake to the surface of the adsorbent. The rapid increase in adsorption capacity in the first 120 min was associated with the dominating diffusion phase. Within that range of contact time, the averaged Pb^2+^ removal percentage increased as much as 29.71%. The slow increase of the adsorption capacity in the contact time was due to the occupied binding sites. Ideally, the adsorption reached an equilibrium where the adsorbate uptake equaled the ones released from the adsorbent [24,36,46]. This state seemed to have been reached after 120 min, where only a slight increase in adsorption capacity was given. Nevertheless, additional experiments used a 12 h contact time for complete equilibrium. In this experiment of the contact time effect, the stock solution pH was 5.5.

Figure 9b presents the pH of the solution after Pb^2+^ removal by the CA–PU film adsorbent. The adsorption capacity and the pH value were correlated. At pH 1–2, the adsorption was relatively lower, possibly because the adsorbent material was damaged by the strong acidity. This was indicated by the darkened color of the film’s visual appearance. The lower adsorption at pH 1–2 can also be attributed to the presence of competing for the H^+^ ions that occupied the binding sites in the adsorbent. Nevertheless, at pH 1–2, the removal percentage of Pb^2+^ was as high as 48.23–51.04%. However, at pH 7, the Pb hydroxide ion (Pb(OH)^−^) species were present in the aqueous system. Hydroxide ions were responsible for heavy metal adsorption, as reported in previous research [47]. 

At the pH range of 6–7, the presence of neutral lead(II) carbonate (PbCO_3_) increased, which according to Utomo et al. [48], can reduce the adsorption capacity. In this research, the optimum condition was obtained at pH 7, which suggests that the electrostatic force was not the only adsorption mechanism. Alternatively, chelation may be responsible for the adsorption mechanism as the N-containing groups introduced into the adsorbents have been known to bind heavy metals through chelation [12,24]. Further pH range was not investigated as it would yield Pb precipitates and obscure the real behavior of Pb^2+^ adsorption on the CA–PU film adsorbent. 

The effect of initial concentration at 12 h contact time and pH 7 can be seen in Figure 9c. Higher initial concentration effectively forced more Pb^2+^ adsorbates to be diffused into the solid phase. As a consequence, an increase in the adsorption capacity could be observed. The initial concentration had a strong linear correlation with the number of adsorbed Pb^2+^ with R^2^ = 0.99721. These results were used for the isotherm studies.

### 3.6. Adsorption Isotherms

**Figure 10:**
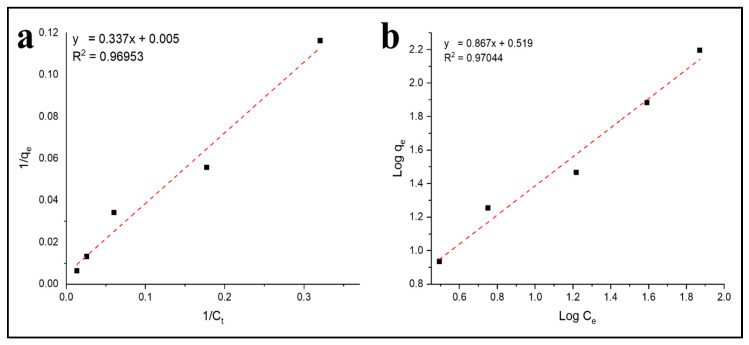
(**a**) Langmuir and (**b**) Freundlich isotherm model for Pb^2+^ adsorption on the CA–PU film adsorbent.

Further study on the adsorbate–adsorbent interaction behavior was conducted with isotherm studies and the result presented in Figure 10 and Table 4. The isotherm adsorption of Pb^2+^ on the CA–PU45 film was studied with the two most common isotherm models, namely the Freundlich and Langmuir isotherm models, which are presented in Figure 10a,b. The model’s fitness was judged based on the given R^2^ value, where a higher R^2^ suggests a higher correlation. The Langmuir isotherm model was constructed based on the homogenous and monolayer adsorption [49]. Meanwhile, the Freundlich isotherm model was based on the heterogeneous binding energy on the adsorbent surface and multilayer adsorption [50]. The linearized forms of the Freundlich and Langmuir isotherm models are presented in Equations (3) and (4), respectively.

(3) $$\frac{1}{q_{e}}=\frac{1}{K_{L}Q_{m}C_{e}}+\frac{1}{Q_{m}}$$

(4) $$\;\;Log\;q_{e}=\frac{1}{n}LogC_{e}+LogK_{F}\;\;$$

where in Equation (2), *K_L_* is the Langmuir isotherm constant (dm^3^/mg) and *Q_m_* is the maximum adsorption capacity (mg/g). Meanwhile, for Equation (3), *K_F_* represents the Freundlich isotherm constant (mg/g) (dm^3^/g), and *n* is the value associated with adsorption intensity. 

Based on the linear regression of the Freundlich and Langmuir isotherm models (Figure 10a,b), it was found that the experimental data were in better agreement with the Freundlich’s isotherm model prediction with a R^2^ value of 0.97044, compared to the Langmuir isotherm with a R^2^ value of 0.96953. The obtained values of the Freundlich and Langmuir isotherm model parameters were presented in Table 4. Since the applicability of the Freundlich isotherm model was high (R^2^ > 0.75), it was noticed that the adsorption intensity parameter (1/n) had a value close to 1 (0.867), which suggests chemisorption dominance [50]. High adsorption intensity was due to the Pb^2+^ adsorption through the chelation mechanism. The higher fitness with the Freundlich isotherm model was due to the different adsorption binding sites and mechanisms involved in Pb^2+^ uptake. The *K_F_* obtained from this study was compared with those reported in the literature (Table 5).

## 4. Conclusions

In summary, preparation of cellulose acetate–polyurethane (CA–PU) by the polycondensation of cellulose acetate with MDI was achieved. The formation of the urethane linkage was confirmed in this study. Furthermore, the improved properties of cellulose acetate with the addition of MDI due to the formation of urethane linkages was established. The characterization results showed improvement in the mechanical, morphology, and thermal properties of the film. SEM images revealed that the addition of MDI increased the contact surface needed for the adsorption. The film tensile properties increased with improved thickness from the AFM studies. The film thermal stability was better than the control sample with the addition of MDI from the TGA and DSC analysis. The mechanical, thermal stability, and adsorption properties increased with the development of the urethane linkage network. The FTIR analysis results showed the formation of N-containing functional groups (NH and NCO) and O-containing functional groups (OH and CO). These functional groups were established as contributors to the Pb^2+^ adsorption properties of the CA–PU film. The heterogeneity of the adsorption system was observed from the isotherm studies in accordance with the Freundlich isotherm model.

## Figures and Tables

**Table 1:** Composition of neat cellulose acetate (CA) and cellulose acetate–polyurethane (CA–PU) film adsorbents.

Label	Cellulose Acetate Powder (g)	1,4-Dioxane (mL)	MDI (% *w*/*w* Cellulose Acetate)
CA	1	10	-
CA–PU15	1	10	15
CA–PU30	1	10	30
CA–PU	1	10	45

**Table 2:** Contact angle measurement of the cellulose acetate (CA) and cellulose acetate–polyurethane (CA–PU) films.

MDI (%)	Droplet Image	Contact Angle	θ
0	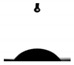	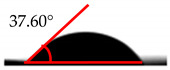	37.60° ± 1.2°
15	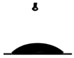	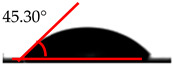	45.30° ± 2.3°
30	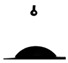	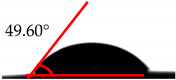	49.60° ± 1.7°
45	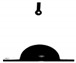	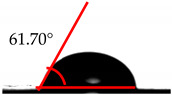	61.70° ± 1.6°

**Table 3:** Tensile strength and elongation percentage of the CA–PUs.

No.	MDI (% *w*/*w*)	Tensile Strength (MPa)	Elongation (%)	Tensile Modulus (MPa)
1	0	17.3 ± 0.3	12.2	140.0 ± 2.1
2	15	17.5 ± 0.2	9.1	123.0 ± 1.5
3	30	20.4 ± 0.6	11.9	168.0 ± 1.2
4	45	24.1 ±0.9	15.6	213.0 ± 1.7

**Table 4:** The obtained values of the isotherm model parameters and coefficient of determination.

Langmuir			Freundlich		
R^2^	*Q_m_* (mg/g)	*K_L_* (dm^3^/mg)	R^2^	*K_F_* (mg/g)(dm^3^/g)	1/n
0.96953	200	0.015	0.97044	2.380	0.867

**Table 5:** Comparison of *K_F_* with previous studies.

No.	Adsorbent	*K_F_* (mg/g) (dm^3^/g)	Citation
1	Chitosan-iron(III) bio-composite beads	0.69	Lapo et al. [51]
2	Chili seeds (Capsicum annuum) waste	0.263	Medellin-Castillo et al. [52]
3	Apple peel-based activated carbon	2.2637	Enniya et al. [12]
4	Albizia lebbeck pods-based powder	1.28	Mustapha et al. [44]
5	NaOH/urea-based graphene oxide/cellulose hydrogel	0.92	Chen et al. [53]
6	Chitosan–pyromellitic dianhydride modified biochar	5.0407	Deng et al. [45]
7	Cellulose acetate–polyurethane Film	2.380	This research
